# Ketogenic Diet Improves Sleep Quality and Daytime Sleepiness in Chronic Migraine: A Pilot Study

**DOI:** 10.3390/neurolint16060091

**Published:** 2024-10-25

**Authors:** Yan Tereshko, Simone Dal Bello, Enrico Belgrado, Cherubino Di Lorenzo, Alice Pittino, Francesca Filippi, Francesca Valdemarin, Christian Lettieri, Gian Luigi Gigli, Annacarmen Nilo, Gaia Pellitteri, Giovanni Merlino, Mariarosaria Valente

**Affiliations:** 1 Clinical Neurology Unit, Department of Head, Neck and Neurosurgery, Udine University Hospital, Piazzale Santa Maria della Misericordia 15, 33100 Udine, Italy; 2 Stroke Unit, Department of Head, Neck and Neurosurgery, Udine University Hospital, Piazzale Santa Maria della Misericordia 15, 33100 Udine, Italy; 3 Neurology Unit, Department of Head, Neck and Neurosurgery, Udine University Hospital, Piazzale Santa Maria della Misericordia 15, 33100 Udine, Italy; 4 Department of Medico-Surgical Sciences and Biotechnologies, Sapienza University of Rome, Polo Pontino, 04100 Latina, Italy; 5 Department of Medicine (DMED), University of Udine, Via Colugna 50, 33100 Udine, Italy

**Keywords:** ketogenic diet, migraine, headache, migraine prevention, low-glycemic index diet, 2:1 ketogenic diet

## Abstract

Aims: The aim of this study is to assess the sleep quality and daytime sleepiness improvement in chronic migraineurs after 6 months of a 2:1 KD (ketogenic diet) and LGID (low-glycemic-index diet). Methods: Twenty-six patients underwent 2:1 KD (11 patients) and LGID (15 patients). PSQI (Pittsburgh sleep quality index) and ESS (Epworth sleepiness scale) were administered at the baseline and the 3-month and 6-month follow-up. MIDAS (Migraine Disability Assessment), HIT-6 (Headache Impact Test 6), migraine frequency (migraine days per month), migraine intensity, BMI (Body Mass Index), FM (Fat Mass), and FFM (Fat-Free Mass) were also assessed. Results: PSQI (F_1.544, 38.606_ = 7.250; *p* = 0.004), ESS (F_1.988, 49.708_ = 9.938; *p* < 0.001), HIT-6 (F_1.432, 35.805_ = 12.693; *p* < 0.001), migraine frequency (F_1.522, 38.041_ = 23.070; *p* < 0.001), migraine intensity (F_1.949, 48.721_ = 18.798; *p* < 0.001), BMI (F_1.274, 31.857_ = 38.191; *p* < 0.001), and FM (F_1.245, 31.134_ = 45.487; *p* < 0.001) improved significantly. The MIDAS (F_1.005, 25.121_ = 3.037; *p* = 0.093) and the FMM (F_1.311, 32.784_ = 1.741; *p* = 0.197) did not improve significantly. The ESS (*p* = 0.712) and PSQI (*p* = 0.776) data at 3-month and 6-month follow-ups did not differ significantly, as well as for migraine frequency, migraine intensity, BMI, FM, and HIT-6. A mild correlation emerged between the mean FM and mean ESS reduction during the 6 months (*r* = 0.497, *p* = 0.010). Conclusions: Six months of LGID and 2:1 KD can improve sleep quality and daytime sleepiness in patients with chronic migraine. The effectiveness on migraine, sleep quality, and daytime sleepiness does not differ significantly between the 3-month and 6-month follow-up periods.

## 1. Introduction

Headaches and sleep disorders are chronic conditions that affect a large portion of the population, posing substantial social and economic burdens [1,2].

Insomnia is a common sleep disorder, often overlooked, occurring in individuals of all ages. Prevalence estimates indicate that one to two-thirds of adults experience symptoms of insomnia, with approximately 10–15% receiving a diagnosis of chronic insomnia disorder [1,3].

Migraine is a primary headache characterized by various combinations of neurological, gastrointestinal, and autonomic disturbances [1,4]. The pathophysiology of migraine is complex, with both clinical and laboratory evidence suggesting that susceptibility can be either genetic or acquired. Individual migraine attacks are often triggered by disruptions in homeostasis, leading to a series of events, including the activation of cortical spreading depression, central and peripheral sensitization, and stimulation of the trigeminovascular pathway. This pathway triggers the release of vasodilatory, pro-inflammatory, and pain-inducing neuropeptides, such as calcitonin gene-related peptides. Chronic migraine is linked to lowered nociceptive thresholds, heightened sensitization, and structural brain changes, such as cortical thinning [5]. Chronic migraine patients experience headaches on at least 15 days per month, with migraine-specific symptoms occurring on at least eight of those days, meeting the diagnostic criteria for migraine [6]. Migraine is a common disorder, affecting 18% of women and 6% of men, while chronic migraine affects 2% of the global population [4]. Interestingly, poor sleep quality is associated with an increased risk of developing migraine, and migraineurs have poorer sleep quality than controls [7]. Moreover, headache frequency is associated with excessive daytime sleepiness [8,9].

The existence of a relationship between migraine and sleep disorders has been known for centuries; however, the exact nature of this association, the underlying mechanisms, and interactions are complex and not fully understood [1,10]. More studies are needed to investigate the use of diet in the treatment of sleep disorders.

The ketogenic diet encompasses any intervention that induces the production of ketone bodies as the sole alternative energy source to glucose. Multiple ketogenic dietary interventions are used in clinical practice: the classic ketogenic diet, modified Atkins diet, low-glycemic-index diet, low-calorie ketogenic diet, very low-calorie ketogenic diet, low-glycemic-index diet, and Mediterranean ketogenic diet. During a ketogenic diet, when external glucose is unavailable, the liver converts fatty acids into ketone bodies—acetoacetate, β-hydroxybutyrate, and acetone. These ketone bodies are then transported to metabolically active tissues such as skeletal muscle, brain, and heart, where they are converted into acetyl-CoA, fueling the Krebs cycle.

In the CKD (classic ketogenic diet), the fat-to-non-fat ratio (carbohydrates and proteins combined) is typically set at 3:1 or 4:1. This means that fat consumption is three to four times higher than the intake of carbohydrates and proteins together, measured in grams [11]. The LGID (low-glycemic index diet), by contrast, is defined by a high fat intake (60%), moderate protein intake (around 30%), and a restricted carbohydrate intake (40–60 g) with a glycemic index below 50, minimizing spikes in blood glucose levels. LGID is particularly beneficial for epileptic patients who struggle with the palatability or tolerability of the ketogenic diet. Although it induces lower levels of ketone body production compared to the classic KD (ketogenic diet), it effectively stabilizes blood glucose levels and can still provide therapeutic benefits [12].

There is evidence that three months of KD might improve the sleep quality of migraine patients [13]; one other pilot study reported that the total sleep time and sleep efficiency increased in chronic migraineurs aged 14–18 years who underwent 3 months of KD therapy [14]. However, no evidence exists of the effect on sleep and daytime sleepiness after 6 months of KD in migraineurs [15].

This study aims to retrospectively evaluate the effectiveness of the 2:1 ketogenic diet and low-glycemic-index diet on sleep quality and daytime sleepiness during a period of 6 months in patients with chronic migraines using validated questionnaires such as the Pittsburgh Sleep Quality Index and Epworth Sleepiness Scale. Additionally, we assessed the effect of these diets on migraine and anthropometric parameters.

## 2. Method

This is a longitudinal retrospective single-center study with prospectively collected data. We performed a retrospective analysis of data from 26 patients diagnosed with chronic migraine who were treated with either the 2:1 KD or the LGID as preventive migraine therapies. These patients were followed for a 6-month period, from January 2020 to July 2022, at our nutritional outpatient clinic in the Clinical Neurology Unit of S. Maria della Misericordia University Hospital, Udine, Italy; the diet was tailored on the patient’s personal preferences and BMI (Body Mass Index). No patient who underwent 6 months of diet was excluded from the analysis. The presence of any comorbidities and pregnancy were not permitted; the missing data excluded the patients from the statistical analysis. The patients underwent neurological and nutritional examinations performed by a neurologist and nutritionist. The data collected comprehended the demographics, migraine intensity (NRS 0–10 scale), migraine frequency (migraine days per month), Migraine Disability Assessment, Headache Impact Test 6, previous and concomitant migraine prophylaxes, symptomatic medication intake per month, weight, BMI, dietary preferences, Fat Mass, and Fat-Free Mass. Fat Mass and Fat-Free Mass were assessed using Bioelectrical Impedance Analysis with the 101 BIA PRO device (Akern, Pisa, Italy); these data were collected at the baseline, 3-month, and 6-month follow-up. After 1 month of diet, the patients were re-evaluated by our nutritionist to adjust the diet to increase its tolerance and adherence. The migraine frequency (migraine days per month), migraine intensity (NRS 0–10 scale), the MIDAS (Migraine Disability Assessment), and the HIT-6 (Headache Impact Test 6) were collected using a headache diary at the baseline at 3 months and 6-month evaluations. The data on migraine frequency and intensity to determine the baseline were collected one month before the initiation of diet therapy. Concomitant migraine preventive therapies (4 patients in therapy with Amitriptyline, 4 patients with Venlafaxine, 1 patient with Lamotrigine, 2 patients with Topiramate, 2 patients with Galcanezumab, and 1 patient with Trazodone) were permitted if these medications were present for at least 3 months before the diet therapy and not modified during the 6 months of the follow-up period; other medications were not permitted. The Epworth Sleepiness Scale and the Pittsburgh Sleep Quality Index scales were administered to the patients at baseline, 3-month, and 6-month follow-up.

### 2.1. Ethical Aspects

This study was conducted in accordance with the Declaration of Helsinki and received approval from the Institutional Review Board of the University of Udine (IRB-DAME, Protocol IRB: 103/2022, approved on 11 July 2022). All patients provided written informed consent for their treatment.

### 2.2. Ketogenic Diet Therapy

The dietary regimens (2:1 KD or LGID) were customized according to each patient’s preferences: Body Mass Index, Fat Mass, and Fat-Free Mass. The protein and fat content in each diet was determined based on anthropometric measurements, Bioelectrical Impedance Analysis, and the patient’s level of daily physical activity. Carbohydrate intake was fixed at 30 g per day and was not adjusted for either diet. Protein amounts were calculated based on FFM (Fat-Free Mass), with conversions to grams considering activity levels and ideal body weight. The LGID was prescribed to patients with a BMI of 25–29.9 kg/m^2^, due to its greater palatability and tolerability; this diet provided a total caloric intake of 1300–1500 kcal/day, with fat content equivalent to the combined total of protein and carbohydrates. Conversely, the 2:1 KD was prescribed for patients with a BMI of 18.5–24.9 kg/m^2^, offering a total caloric intake of 1600–2300 kcal/day, with fat content equal to double the sum of protein and carbohydrates.

### 2.3. Endpoints

#### 2.3.1. Primary Endpoint

The effect of the diet on sleep quality and daytime somnolence during the 6 months of follow-up was determined using the PSQI (Pittsburgh sleep quality index) and the ESS (Epworth sleepiness scale) scales. Moreover, the modifications of migraine intensity (NRS 0–10 scale), frequency (migraine days per month), MIDAS, and HIT-6 were also evaluated.

#### 2.3.2. Secondary Endpoint

To assess the correlation between the mean PSQI and ESS reduction with the mean BMI, HIT-6, MIDAS, FM (Fat Mass), FFM, migraine intensity, and migraine frequency 6-month reduction.

### 2.4. Data and Statistical Analysis

Since this was a pilot study, the power of this study was not calculated; a descriptive analysis of the study population’s main features was performed using mean ± SD for continuous variables and absolute and relative frequencies for categorical variables. A Shapiro–Wilk test was used to assess the normal data distribution. A repeated measure ANOVA was performed to investigate the changes in migraine intensity (NRS 0–10 scale), migraine frequency (days per month), MIDAS, HIT-6, PSQI, ESS, BMI, FM, and FFM at the baseline (T0) at 3-month (T1) and 6-month (T2) follow-ups. Because Mauchly’s test of sphericity was significant, we used the Greenhouse–Geisser correction. The Bonferroni post hoc test was used to compare the means at different follow-up times. Correlation analysis was performed with Spearman’s test. All analyses used Stata/SE (version 15.1, StataCorp, College Station, TX, USA) for Mac OS. The statistical significance levels were set at *p* < 0.05.

## 3. Results

Twenty-six patients with chronic migraine were available to perform the statistics. Twenty-two were female (85%), and four were male (15%). The age was 46.5 ± 13.6 years, and the mean BMI was 24.6 ± 4.8. The mean number of previous prophylaxes was 3.0 ± 2.3; most were on longstanding preventive therapies during this study (85%). Detailed demographics are shown in Table 1.

**Figure 1:**
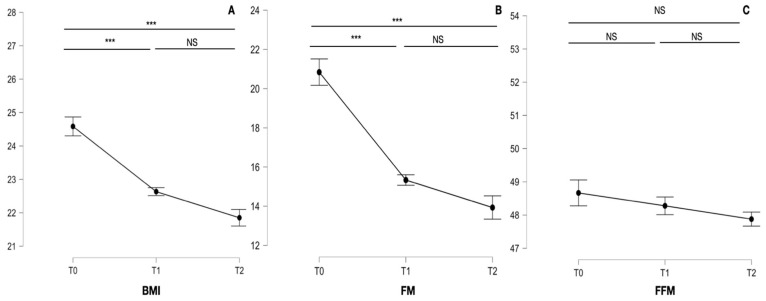
Graphs refer to the BMI (**A**), FM (**B**), and FFM (**C**) of the patients studied at baseline (T0), at 3-month (T1), and 6-month (T2) follow-up. We marked statistically significant differences with the symbol ***, while non-statistically significant differences were indicated as NS.

**Figure 2:**
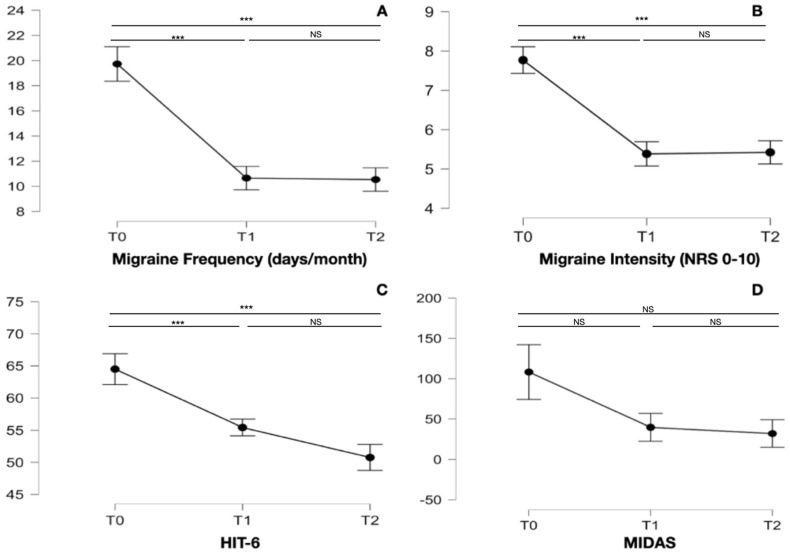
Graphs referred to migraine frequency (**A**), migraine intensity (**B**), HIT-6 (**C**), and MIDAS (**D**) of the 26 patients studied at the baseline (T0), at 3-month (T1), and 6-month (T2) follow-up. We marked statistically significant differences with the symbol ***, while non-statistically significant differences were indicated as NS.

During the 6 months of ketogenic diet therapy, there was a significant reduction in FM (F_1.245, 31.134_ = 45.487; *p* < 0.001) and BMI (F_1.274, 31.857_ = 38.191; *p* < 0.001), while the FFM remained unchanged (F_1.311, 32.784_ = 1.741; *p* = 0.197); see Figure 1. The migraine frequency (F_1.522, 38.041_ = 23.070; *p* < 0.001) and the migraine intensity (F_1.949, 48.721_ = 18.798; *p* < 0.001) improved significantly. The migraine disability determined with the HIT-6 (F_1.432, 35.805_ = 12.693; *p* < 0.001) had significant reduction during the 6 months of treatment; the MIDAS (F_1.005, 25.121_ = 3.037; *p* = 0.093) did not improve significantly (see Figure 2). There was a significant reduction in BMI, FM, migraine intensity, migraine frequency, and HIT-6 during the first three months of diet; however, during the last three months of follow-up, these variables did not change significantly. The detailed data and the Bonferroni post hoc test analysis for these variables are shown in Table 2.

**Figure 3:**
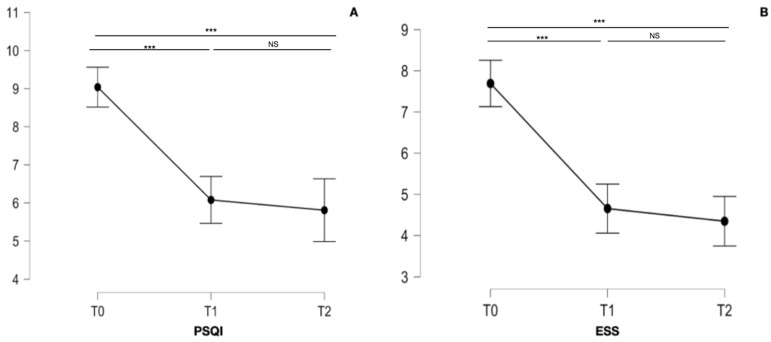
Graphs refer to PSQI (**A**) and ESS (**B**) of the 26 patients studied at the baseline (T0), at 3-month (T1), and 6-month (T2) follow-up. We marked statistically significant differences with the symbol ***, while non-statistically significant differences were indicated as NS.

The sleep quality and the daytime somnolence improved significantly, as shown by the PSQI (F_1.544, 38.606_ = 7.250; *p* = 0.004) and the ESS (F_1.988, 49.708_ = 9.938; *p* < 0.001), respectively; see Table 3, Table 4 and Table 5 and Figure 3 for the detailed statistics. The PSQI and ESS significantly improved during the first three months of diet; the comparison between the 3-month and the 6-month follow-up was not significant. There was a moderate positive correlation between the mean ESS reduction and the mean FM reduction over the six months of follow-up (r = 0.497, *p* = 0.010); this was not found for the mean PSQI reduction and the mean FM reduction. Moreover, the mean ESS and PSQI reductions did not correlate with the mean BMI, FFM, MIDAS, HIT-6, frequency, and intensity reductions.

The side effects of the LGID and 2:1 KD diets were mild and consisted of constipation, bloating, or diarrhea. The details are shown in Table 5.

## 4. Discussion

The relationship between migraine and insomnia is documented and has been described by many authors, although the pathophysiology of both conditions still presents several unclear points. There is evidence that migraineurs have higher PSQI scores compared to healthy controls and that this effect is greater in patients with a chronic condition rather than episodic [16].

The rationale for applying KD in insomnia and migraine derives from the hypothesis that by improving the energy availability of the neuronal cell, the efficiency of both migraine and sleep can be increased. A dysfunctional mitochondrion could be at the root of both insomnia [17] and migraine [18]. Furthermore, insomnia and migraine could also be mutually influenced in a bidirectional relationship: nocturnal migraine attacks could compromise sleep quality, while insomnia could, in turn, lower pain thresholds, thus exacerbating migraine. It is also known that the same sleep disturbance would worsen mitochondrial function, further exacerbating this vicious cycle [19]. This vicious cycle could be improved by applying the ketogenic diet, which, through improved mitochondrial energy efficiency ensured by ketone bodies compared to carbohydrates [20], allows for better neuronal function with benefits on both sleep and migraine. However, separating the effect of the KD solely on insomnia or migraine proves impossible, given the close correlation between the two conditions.

In this study, LGID or KD 2:1 diets were applied for 6 months to a population of subjects with chronic migraine. The studied population is predominantly female, which aligns with the epidemiology of migraine [4]. Few studies in the literature have applied the ketogenic diet to patients with chronic migraines. In 2021, Bongiovanni et al. conducted a study using either a low-carbohydrate ketogenic diet or a very low-carbohydrate ketogenic diet on 23 patients with refractory chronic migraine and medication-overuse headaches for 3 months. They observed significant benefits, including an 80% reduction in headache frequency, a notable decrease in pain intensity, and reduced use of symptomatic medications. Similar to our findings, Bongiovanni et al. also demonstrated a reduction in mean BMI; however, they did not assess body composition. Additionally, no assessments were made regarding sleep quality or daytime sleepiness scales [21].

In 2022, Lovati et al. published a study in which thirteen patients with refractory chronic migraine were treated for only 3 weeks with a modified Atkins diet, while eight patients followed a low carbohydrate diet. Both diets were effective in reducing headache frequency, intensity, symptomatic medication use, and BMI, although the MAD showed greater benefits compared to the LCD. However, the groups were unbalanced in terms of age, with the LCD group being older. This study was later expanded to include 26 refractory chronic migraine patients treated with MAD and 5 with LCD. In this expanded cohort, the ketogenic diet (MAD) demonstrated a clear superiority over LCD in improving headache outcomes. However, neither study assessed the impact of these interventions on sleep quality or daytime sleepiness [22].

In 2023, Tereshko et al. conducted a study applying a 2:1 ketogenic diet or low-glycemic-index diet for 3 months to 60 patients, including both chronic and high-frequency migraine sufferers. They observed improvements in migraine intensity, frequency, MIDAS, and HIT-6 scores, as well as reductions in Fat Mass, weight, and BMI in both groups [12]. Later in 2023, Tereshko et al. treated 76 high-frequency and chronic migraine patients with three different types of KD (2:1 KD, LGID, and Very low-calorie ketogenic diet) over a 3-month period, demonstrating benefits for both headache and fatigue [23]. However, neither of these studies assessed sleep quality or daytime sleepiness scales.

In 2024, Olitivo et al. applied a Mediterranean ketogenic diet for 8 weeks to 23 patients with chronic migraine, demonstrating improvements in both migraine symptoms (reduced pain intensity and headache frequency) and body composition, as assessed by Bioelectrical Impedance Analysis (BIA). However, this study did not evaluate the effects on sleep quality or daytime sleepiness [24].

Only in 2023 did Merlino et al. assess the improvement in sleep quality and daytime sleepiness in 70 patients with episodic and chronic migraine after 3 months on a ketogenic diet with a 2:1 or 1:1 ratio [13]. After that, recently, in 2024, Oasca et al. published preliminary data from a study applying the classic ketogenic diet, with ratios up to 2:1, in seven adolescent patients with chronic migraine. The findings suggested potential benefits for migraine, quality of life, and sleep efficiency. Regarding sleep, actigraphic data indicated possible stabilization, while polysomnographic data showed a slight increase in total sleep time and sleep efficiency, along with a reduction in nighttime awakenings. Additionally, there was a trend toward decreased NREM stage 1 sleep and increased REM sleep. However, the number of patients recruited in this study is quite small, and the population examined consists exclusively of adolescents [14]. The differences in the application of ketogenic dietary regimens between adults and children remain poorly defined, as no comparative studies have been conducted yet, despite the undeniable metabolic differences between these two populations.

Our study is the first to apply a ketogenic dietary regimen for 6 months in chronic migraine patients, specifically evaluating improvements in sleep quality and daytime sleepiness. However, the benefits observed at 3 months were not significantly different from those at 6 months, either in terms of daytime sleepiness and sleep quality or in relation to headache improvement. This is probably because the effect of the KD becomes evident in the early months of the diet, reaching a plateau of effectiveness thereafter. This raises the question of whether the dietary regimen could be limited to 3 months, as extending it to 6 months does not appear to offer additional benefits. However, it is possible that significant differences may appear with larger samples. It is also important to note that such a diet, particularly in the context of migraine, is typically followed for short periods, usually not exceeding 6 months. This is to avoid the onset of side effects such as muscle cramps, constipation, hyperlipidemia, and gallstones [11].

Fatigue has not been evaluated in our study, but it has recently been shown to improve in migraine patients treated with KD [23], which could be partly correlated with the improvement in sleep quality.

The literature also includes studies applying the ketogenic diet to other primary headache models. In 2018, Di Lorenzo et al. implemented an Atkins ketogenic diet (AKD) for 3 months in 18 patients with chronic cluster headache, demonstrating a significant reduction in attack frequency [25]. This type of headache, attributable to different pathophysiological mechanisms compared to migraine, is linked to hypothalamic activation, which is also a common mechanism in insomnia. Future research could explore sleep quality in patients with cluster headaches undergoing treatment with a ketogenic diet.

Our study revealed a weak correlation between the reduction in Fat Mass and the decrease in Epworth Sleepiness Scale scores. This could be because reducing Fat Mass may decrease the risk of obstructive sleep apnea, leading to improvements in daytime sleepiness [26]. Conversely, long-term improvements in sleep quality may help prevent weight gain, suggesting a bidirectional relationship between sleep and obesity [27].

### Study Limitations

Our study has some limitations. First, the relatively small sample and the absence of polysomnographic data. Since this is a single-center study, there could be a bias in patients’ selection, implying that the sample could not represent the entire population of chronic migraineurs. Moreover, in our study, as in all others in the literature, patients who discontinued the diet before the 6-month mark due to ineffectiveness or side effects were not included in the statistical analyses. Consequently, the treatment may currently be overestimated.

## 5. Conclusions

Three months of LGID and 2:1 KD can improve sleep quality and daytime sleepiness in chronic migraine patients, independent of improvements in BMI and migraine parameters. The benefits observed at 3 months were not significantly different from those at 6 months, both in terms of daytime sleepiness and sleep quality, as well as headache improvement. This suggests that the effects of the KD may manifest early in the diet, reaching a plateau thereafter. This raises the question of whether the dietary regimen could be effectively limited to 3 months, as extending it to 6 months does not seem to provide additional benefits. However, it is possible that significant differences might emerge with larger sample sizes.

## Figures and Tables

**Table 1:** Characteristics of the chronic migraineurs treated with ketogenic diet (low-glycemic index diet or 2:1 ketogenic diet).

Variable	(Mean ± SD or *n*, /%)
Number of patients	26
Age	46.5 ± 13.6
Female Sex	22 (85%)
BMI	24.6 ± 4.8
Fat Mass	20.8 ± 8.3
Fat-Free Mass	48.7 ± 8.6
Years of having migraines	21.8 ± 16.4
Previous migraine prophylaxis	3.0 ± 2.3
Concomitant prophylaxys	18 (69%)
Symptomatic medication intake/month	14.4 ± 9.3
MIDAS	108.3 ± 237.7
HIT-6	64.5 ± 8.1
Migraine days/month frequency	19.7 ± 8.8
Migraine Intensity (NRS)	7.8 ± 1.1
LGID	15 (58%)
2:1 KD	11 (42%)
PSQI > 5	16 (62%)
ESS > 10	7 (27%)

**Table 2:** Anthropometric characteristics and migraine data at baseline, 3-month, and 6-month assessments of the 26 patients studied (mean and SD). The means of these variables were compared with the Bonferroni post hoc test. We highlighted in bold the statistically significant values of *p*. Legend: FM, Fat Mass; FFM, Fat-Free Mass; BMI, Body Mass Index; * T0 vs. T1; ** T0 vs. T2; *** T1 vs. T2.

	Baseline	3-Month Follow-Up	6-Month Follow-Up	*p* (Post Hoc Comparison)
BMI	24.6 ± 4.8	22.6 ± 3.9	21.9 ± 3.2	*,** <0.001, *** 0.057
FM	20.8 ± 8.3	15.3 ± 6.4	13.9 ± 4.8	*,** <0.001, *** 0.073
FFM	48.7 ± 8.6	48.2 ± 8.1	48.9 ± 8.1	* 0.697, ** 0.204, *** 0.697
Migraine intensity	7.8 ± 1.1	5.4 ± 2.5	5.4 ± 2.5	*,** <0.001, *** 0.932
Migraine frequency	19.7 ± 8.8	10.7 ± 10.1	10.5 ± 10.2	*,** <0.001, *** 0.941
MIDAS	108.3 ± 237.7	39.8 ± 36.5	32.2 ± 37.0	* 0.099, ** 0.089, *** 0.823
HIT-6	64.5 ± 8.1	55.4 ± 14.5	50.7 ± 17.2	* 0.006, ** <0.001, *** 0.298

**Table 3:** Data of the PSQI and ESS variables (mean and SD). The means of these variables were compared with the Bonferroni post hoc test. We highlighted in bold the statistically significant values of *p*. Legend: * T0 vs. T1; ** T0 vs. T2; *** T1 vs. T2.

	Baseline	3-Month Follow-Up	6-Month Follow-Up	*p*
ESS	7.7 ± 3.3	4.7 ± 3.1	4.3 ± 3.1	* 0.001, ** <0.001, *** 0.712
PSQI	9.0 ± 3.8	6.1 ± 3.6	5.8 ± 4.5	* 0.006, ** 0.004, *** 0.776

**Table 4:** Results from Spearman’s correlation test. There was a significant correlation between the mean FM reduction and the mean ESS reduction in these 6 months of follow-up. We highlighted in bold the statistically significant values of *p*.

	6-Months Mean PSQI Reduction	6-Months Mean ESS Reduction
6-month Mean BMI reduction	r = 0.138, *p* = 0.500	r = 0.332, *p* = 0.097
6-month Mean FM reduction	r = 0.268, *p* = 0.185	**r = 0.497, *p* = 0.010**
6-month Mean FFM reduction	r = 0.018, *p* = 0.930	r = 0.233, *p* = 0.252
6-month Mean MIDAS reduction	r = 0.003, *p* = 0.988	r = 0.019, *p* = 0.925
6-month Mean HIT-6 reduction	r = 0.264, *p* = 0.193	r = 0.046, *p* = 0.825
6-month Mean Frequency reduction	r = 0.096, *p* = 0.643	r = 0.029, *p* = 0.890
6-month Mean Intensity reduction	r = 0.084, *p* = 0.682	r = 0.109, *p* = 0.597

**Table 5:** Side effects reported by our patients, reported by the type of ketogenic diet.

	LGID	2:1 KD
Constipation	4/15 (26%)	2/11 (18%)
Bloating	3/15 (20%)	3/11 (27%)
Diarrhea	1/15 (7%)	0/11 (0%)

## Data Availability

The datasets used and/or analyzed during this study are available from the corresponding author upon reasonable request from any qualified investigator.

## Abbreviations

KD	ketogenic diet
CKD	classic ketogenic diet
LGID	low-glycemic-index diet
VLCKD	very low-calorie ketogenic diet
LCKD	low-calorie ketogenic diet
MAD	modified Atkins diet
VLCnKD	very low-calorie not ketogenic diet
LCD	low-carbohydrate diet
FM	Fat Mass
FFM	Fat-Free Mass
BMI	Body Mass Index
MIDAS	Migraine Disability Assessment
BIA	Bioelectrical Impedance Analysis
HIT-6	Headache Impact Test 6
SD	standard deviation
CI	confidence interval
NRS	Numeric Pain Rating Scale
ESS	Epworth Sleepiness Scale
PSQI	Pittsburgh Sleep Quality Index
MOH	medication-overuse headache
